# Editorial: Promoting health equity during a pandemic: approaches to address vaccination burden and health inequities amongst under-served populations in U.S. and Mexico

**DOI:** 10.3389/fpubh.2023.1240391

**Published:** 2023-07-07

**Authors:** Hilda Davila, Cecilia Rosales, Maria Gudelia Rangel Gomez

**Affiliations:** ^1^Instituto de los Mexicanos en el Exterior, Mexico City, Mexico; ^2^University of Arizona, Tucson, AZ, United States; ^3^El Colegio de la Frontera Norte, Tijuana, Mexico; ^4^United States - Mexico Border Health Commission, Tijuana, Baja California, Mexico

**Keywords:** COVID-19, vaccination, under-served populations, health equity, community health

COVID-19 exacerbated existing health inequities and led to unique ones in vulnerable and historically underserved populations. This edition of *Frontiers* documents the multiple challenges associated with COVID-19 and efforts to ameliorate them among diverse populations that reside in the US Mexico border region and beyond.

The border between Mexico and the United States is a complex space where the unique social and economic context of the region impacts the health, economy and other social determinants which contribute to significant health inequities among the diverse populations that inhabit the region. One consequence of the COVID-19 pandemic was to further aggravate these existing health inequities. For example, Muñiz-Salazar et al. describes how the pandemic interrupted ongoing efforts to detect TB and provide treatment for tuberculosis in the state of Baja California. It also documented how the pandemic limited existing TB services and required service providers to develop and adapt new strategies to detection and treatment such as relying on telemedicine. In another study, Wagler et al. reveals the impact of the pandemic on specific social determinants of health that lead to high-risk behaviors such as unhealthy diets.

The COVID-19 pandemic not only aggravated existing health inequities but also led to unique ones as well. Several of the articles in this Research Topic remind us that technical solutions, such as vaccines, need to account for the social context within which they are implemented to ensure their widespread adoption. A case study in Tucson, Arizona of workers at beauty salons and auto shops provides solid information in addressing this topic in the article by Moreno Ramírez et al.. It also reminds us of the centrality of essential workers to the functioning of society and the importance of integrating the workplace into the public health response. Studies in Miami-Dade County and Arizona also highlight that social determinate of health, such as migration, that are commonly found on the border are not limited to the geographical region. Bastida et al. and Nuño T. et al. provide evidence of the causes of vaccine hesitancy and acceptance allowing public policymakers to consider the impact of health messages and intervention programs anchored by scientific evidence and the authenticity of the messenger. Finally, Harvey-Vera et al. centers the experience of people who inject drugs and how social stigma and discrimination that they experience along with the limited access to health services complicates COVID-19 vaccination efforts. It reminds us of the importance of accounting for the heterogeneity within populations and the need to tailor interventions to a variety of life circumstances.

Migration is another social determinate of health that plays a prominent role in public health efforts in the border region and beyond. The intense mobility that is a trait at the border posed the challenge of detecting COVID-19 infection among migrant populations as lack of transparency in information and lack of institutional coordination are evidenced by Rangel Gómez, Varela, et al.. Cruz Piñeiro and Ibarra report results from interviews of Central American and Caribbean immigrants stranded in four border cities in Mexico. The experience of these immigrants speaks to the impact of over-crowded shelters and lack of access to services affected the mental, social, and economic health of this population from a diverse cultural and economic perspective. Also deported Mexican migrants faced an increased risk of COVID-19 infections due to a lack of vaccines and unhealthy conditions in detention settings in the US as is described by Martínez-Donate et al..

Several articles in this Research Topic address the unique mental health impacts of the COVID-19 pandemic. Morales Chainé et al. addresses the mental health symptoms that impacted the population suffering from COVID-19 and the need to target specific populations such as women with psychological care. Furthermore, Jaramillo et al. attests to the centrality of mental health issues to the pandemic. The article not only contributes evidence from the 794 screenings but also documents a unique strategy of bi-directional monitoring among non-specialized health personnel at the Health Windows and Mobile Health Units for the immigrant population that will allow the promotion and early care for mental health beyond the COVID pandemic. The impact of COVID-19 on the healthy development of adolescents is still an interrogation mark, evidence is key to understanding how long COVID-19 plays a role in depression and anxiety, and other acute and long-term illnesses. Nuño V. L. et al. describes the procedure to gather evidence that in the future may inform policy in a multifaceted study of adolescents 12 to 17 years through intersectionality theory.

The articles in this Research Topic not only highlight the impact of the COVID-19 pandemic on the health of different populations in the border region and beyond but also highlight the challenges and limitations of public health efforts to ensure access to effective messages, services, and interventions. Migrant communities are fluid and require cultural humility from service provides and public health officials to create credible and culturally sensitive information as  Rangel Gómez, Cruz-Piñeiro, et al. concludes. Lower health insurance rates limited English proficiency, and fear of deportation and discrimination, among others, require public health organizations to adopt non-traditional engagement and outreach approaches such as Ventanillas de Salud (VDS) and Mobile Health Units (MHU) to engage effectively with these populations. Several articles in this Research Topic highlight factors, such as availability and health regulations, that negatively impacted the health of those populations with comorbid conditions such as diabetes, hypertension, and obesity. Infante et al. calls for an inclusive policy that, together with the assistance of civil society organizations, helped migrants to overcome barriers to service and make public health institutions more accessible.

To address some of these limitations, the Centers for Disease Control and Prevention (CDC) funded several multi-year cooperative agreements with existing national networks serving immigrant communities across the United States with the goal of improving health promotion and response activities with limited English proficiency (LEP) Latino essential workers, their families, and the communities where they live. One such cooperative agreement, entitled “*Improving Clinical and Public Health Outcomes through National Partnerships to Prevent and Control Emerging and Re-Emerging Infectious Disease Threats*” fostered a partnership among the Mexican Section of the USMBHC, the Latino Commission on AIDS (LCOA) and Alianza Americas (AA) to integrate their existing infrastructure and relationships with these communities into the COVID-19 response efforts. Rosales et al. describes the efforts of the Mobile Health Units (MHU) that formed and essential part of this cooperative agreement. Their work was guided by three strategies: disseminate and adopt; inform and adapt; and target and train; with the aim of improve health promotion and development of healthcare strategies as a direct response to health emergencies such as the COVID-19 pandemic.

Collaborative approaches to support transborder communities during the COVID-19 pandemic were put in place all along the border region to address the health needs with proper communication, coordination, and collaboration as described by Jiménez and Kozo. New collaborative models such as Ventanillas de Salud and Mobile Health Units reveal the importance of networks that allow putting forward preventive health activities for historically underserved populations as Rangel Gómez, Salazar, et al. describes.

Mobile Outreach Vaccination and Education for Underserved Populations (MOVE UP) as well as Mobile Health Units institutionalized a network of collaboration to bridge the health equity gap for disadvantaged populations such as American Indians, Hispanics, and Blacks as is the case in Arizona. Fingesi et al. examines the need to analyze policy interventions to inform and evaluate the impacts of perceptions and experiences of COVID-19.

Latinx populations and immigrant communities experienced higher rates of infection, were overrepresented in essential jobs and were often excluded from government assistance programs such as unemployment benefits and health insurance subsidies or coverage. Furthermore, limitations of public health institutions to effectively address factors such as limited English proficiency, rural locations, and other structural barriers to health complicated access health information, interventions, and care during the COVID-19 pandemic. This issue of *Frontiers* not only identifies health impacts of COVID-19 but helps to build an evidence-base for diverse strategies to improve access to culturally competent health services for historically underserved populations. If translated into practice, the collaborative efforts and models discussed in this Research Topic will serve to build a more inclusive Public Health infrastructure to address endemic health inequities as well as better prepare society for the next pandemic.

## Author contributions

All authors listed have made a substantial, direct, and intellectual contribution to the work and approved it for publication.

